# Emergency microsurgery for patients with soft tissue, skull, and dura complex defects after trauma: a case report 

**DOI:** 10.1080/23320885.2019.1691922

**Published:** 2019-12-03

**Authors:** Masayuki Okochi, Yuzo Komuro, Kazuki Ueda

**Affiliations:** Department of Plastic and Reconstructive Surgery, Teikyo University, Tokyo Japan

**Keywords:** scalp, dura, reconstruction, free flap, emergency

## Abstract

We performed two emergency microsurgical dura, skull, and scalp complex reconstructions. We used the rectus abdominis flap with free fascia lata and the anterolateral thigh flap with vascularized fascia lata. To achieve good postoperative result, reconstruction should be performed before meningitis or wound infection.

## Introduction

Reconstruction of scalp, skull, and dura complex defects after trauma is challenging, as it requires proper dura reconstruction without cerebrospinal fluid leakage and infection. We have reconstructed scalp, bone, and dura composite defects caused by tumor resection, exposure of artificial skull, and major trauma. Successful microsurgery for patients with large scalp defect has been performed using latissimus dorsi myocutaneous flap [1,2], rectus abdominis myocutaneous flap [3], vastus lateralis muscle flap [4], and anterolateral thigh flap [5,6]. However, there are no guidelines for when and how reconstruction should be performed after major trauma. We believe that the reconstructed dura should be covered with well-vascularized, bulky tissue to prevent cerebrospinal fluid leakage and infection after traumatic wounds. In two cases reported below, we performed emergency reconstruction of the scalp, soft tissue, and dura using the rectus abdominis flap with free fascia lata and the anterolateral thigh flap with vascularized fascia lata. We herein describe our operative procedure.

## Case report

### Case no. 1: a 23-year-old man

The patient was referred to our hospital for treatment of a wide contusion wound caused by a traffic accident (Figure 1(a)), in which the patient’s car collided with a heavy-duty truck. He had a 14 × 5 cm contusion wound in the frontal region. His consciousness level was E1V1M4 (Glasgow Coma Scale). The CT and X-ray findings suggested that the depth of his wound reached brain tissue (Figure 1(b)). Emergency surgery was started four hours after his arrival under general anesthesia. Debridement of the skull, dura, and brain was carefully performed by a neurosurgeon. The wound was contaminated with stone, sand, and pieces of glass. All bone fragments and foreign bodies were removed, and irrigation of the wound was performed. After debridement, the patient had a 14 × 12 cm skull defect and a 7 × 7 cm dura defect. Dura reconstruction using free fascia lata was also performed by the neurosurgeon. An 8 × 8 cm fascia lata was harvested from the left thigh and then fixed to the edge of the dura using 4-0 Nurolon sutures (Figure 1(c)). A 15 × 8 cm left rectus abdominis myocutaneous flap was also harvested (Figure 1(d)). The reconstructed dura was covered with the rectus abdominis muscle, and a skin paddle was fixed to the soft tissue defect (Figure 1(e)). Superficial temporal artery and vein were used as recipient vessel. Vascular anastomosis was performed using 9-0 nylon suture. The postoperative course was uneventful. There was no cerebrospinal fluid leakage, epidural abscess, or surgical site infection. The patient’s general condition was good, and his consciousness level gradually improved. He started to move his extremities three weeks postoperatively and recovered consciousness five weeks postoperatively. Twelve weeks after surgery, he was discharged from our hospital without disability. Cranioplasty was performed ten months after the primary surgery. A customized hydroxyapatite block skull (Hoya Technosurgical co, Tokyo, Japan) was placed through the incision from the primary surgery (Figure 1(f)). Six months postoperatively, an 80 × 140 mm tissue expander (PMT corporation, MN, USA) was also inserted. After four months, 350 ml of expansion had been inserted and the rectus abdominis flap skin paddle was removed (Figure 1(g,h)).

Figure 1.(a) Preoperative view. A 14 × 5 cm contusion wound was observed in the frontal region. The wound was contaminated with sand, stone, and small pieces of glass. (b) CT findings. The depth of the wound reached brain tissue. (c) After dura reconstruction. Dura reconstruction was performed using an 8 × 8 cm fascia lata. (d) A 15 × 8 cm sized left rectus abdominis myocutaneous flap was harvested. (e) Postoperative view of forehead, soft tissue and dura reconstruction. The soft tissue was repaired using a skin paddle from the rectus abdominis myocutaneous flap. (f) Cranioplasty was performed ten months after primary surgery. A customized hydroxyapatite block was inserted into the skull defect. (g) Six months postoperatively, an 8 × 14 cm tissue expander was inserted under the scalp. This figure shows four months after tissue expander insertion. (h) Two years after tissue expander removal. The skin paddle made from the rectus abdominis flap was completely removed.

**Figure:**
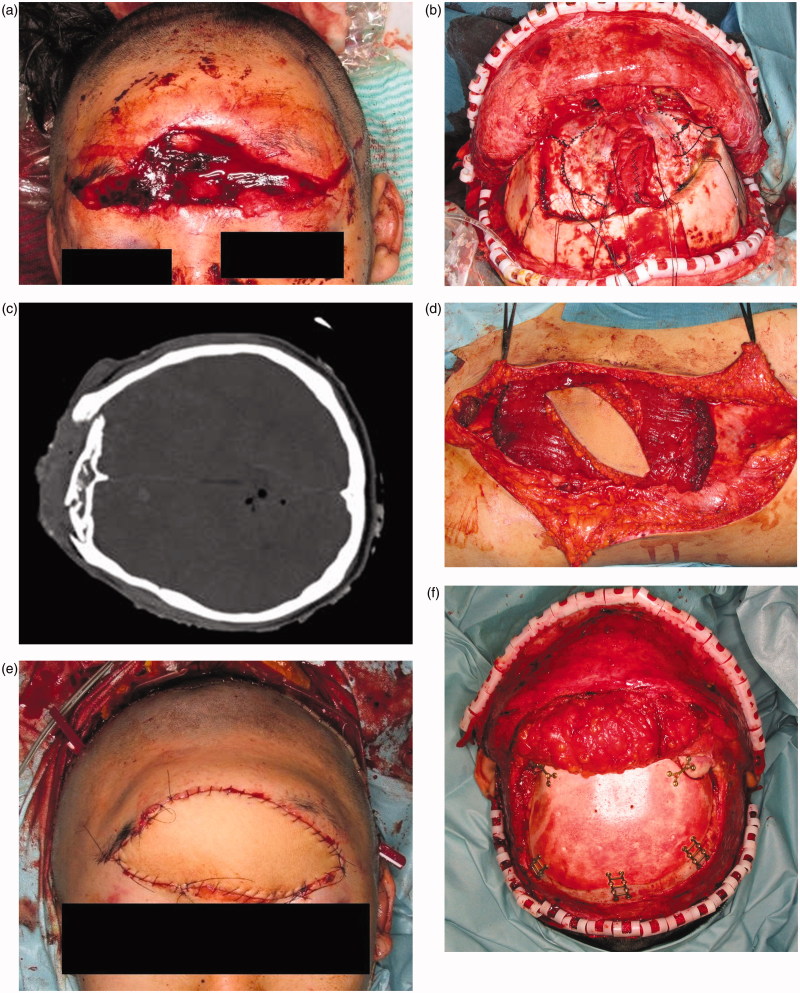

**Figure:**
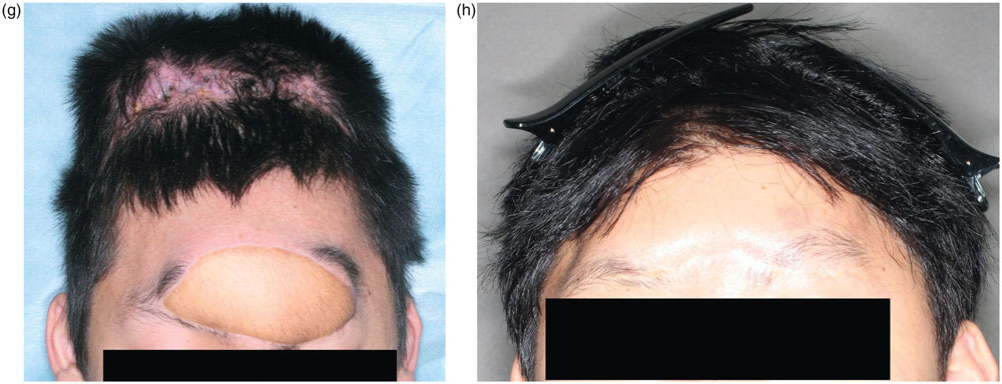

### Case no. 2: a 38-year-old man

**Figure 2.**
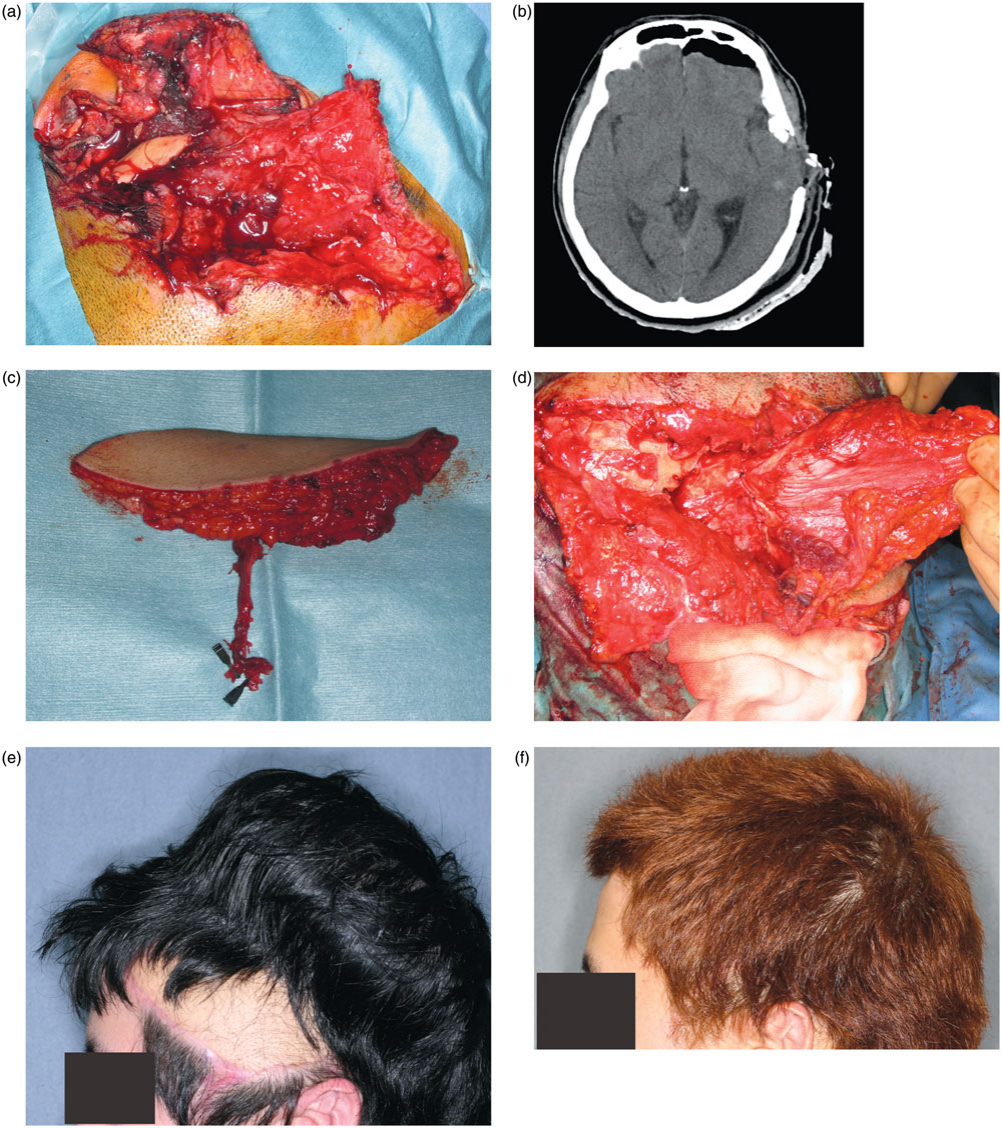
(a) The patient had a large defect (including the soft tissue, scalp, and dura) in the left temporal region. The wound was contaminated with sand, pieces of metal, and machine lubricant. (b) CT findings suggest that the depth of the wound reached the brain tissue. (c) A 15 × 8 cm anterolateral thigh flap with 8 × 8 cm of fascia was harvested. (d) Vascularized fascia was fixed to the edge of the dura. (e) A 70 × 120-cm tissue expander was inserted under the scalp. Preoperatively, 270 ml of expansion was achieved. (f) Two years after final surgery. The skin paddle made from the anterolateral thigh flap was completely removed.

The patient was referred to our hospital with a wide contusion wound in the left temporal region of the scalp caused by an industrial machine. There was a 12 × 8 cm scalp and soft tissue defect on his left temporal scalp, and wound was contaminated with sand, small pieces of metal, machine lubricant and skull fragments (Figure 2(a)). His consciousness level was E2V2M5 (Glasgow Coma Scale). The CT and X-ray findings suggested that the depth of his wound reached brain tissue (Figure 2(b)). Emergency surgery was stared five hours after his arrival. Skull fragments and all foreign bodies removed and debridement of dura and brain was carefully performed by a neurosurgeon. After debridement, an 8 × 7 cm skull defect and a 6 × 6 cm dura defect were observed. During debridement, a 15 × 8 cm anterolateral thigh flap with an 8 × 8 cm fascia lata was harvested (Figure 2(c)). Dura reconstruction was performed by the neurosurgeon and a plastic surgeon. The fascia was fixed to the dura using 4-0 Nurolon sutures (Figure 2(d)). The lateral circumflex femoral artery and vein were anastomosed to the superficial temporal artery and vein. Postoperative course was uneventful. The patient’s general condition was good, and there was no cerebrospinal fluid leakage, epidural abscess, or surgical site infection. Two days postoperatively, he recovered consciousness. Two weeks after surgery, he was discharged from our hospital without disability. Six months postoperatively, a 70 × 120 cm tissue expander (PMT Corporation) was inserted. Four months later, 270 ml of expansion had been achieved and the anterolateral thigh flap skin paddle was removed (Figure 2(e)). Postoperatively, patient did not have any handicap or disability (Figure 2(f)).

## Discussion

Successful emergency microsurgery for patients with a major, complex wounds in the head and neck region has been reported [1–6]. In particular, microsurgery is found to be effective for repairing avulsion wounds. [7–11]. For scalp replantation, early microsurgery is recommended. In our cases, we have performed reconstruction of scalp, bone, and dura composite defects caused by trauma. However, there are no guidelines for when this type of reconstruction after trauma should be performed, and the timing is particularly unclear for soft tissue and dura reconstruction. For reconstruction of a deep, wide scalp wound, the free flap transfer is a useful alternative because reconstructed dura should be covered with well-vascularized tissue. The latissimus dorsi myocutaneous flap [1,2], rectus abdominis myocutaneous flap [3], vastus lateralis muscle flap [4], and anterolateral thigh flap [5] are all recognized as workhorse flaps for wide scalp reconstruction. On the other hand, some authors have reported effective scalp reconstruction using a local flap [12,13], although this method is limited by the depth of the reconstruction site. For deep, complicated wounds, if the reconstructed dura is not covered with well-vascularized, bulky tissue, postoperative CSF leakage may occur. Postoperative leakage of cerebrospinal fluid can lead to wound infections, epidural abscesses, or meningitis [14–20]. Daudia *et al.* reported that 19% of post-traumatic CSF leakage cases resulted in meningitis [14], and meningitis is associated with an 8.9%–10% mortality rate [15,16]. For lower leg reconstruction, Godina *et al.* reported that microsurgical reconstruction with tissue transfer should be performed within 3 days after trauma to avoid scar formation around the recipient artery [21]. In their study, a higher rate of flap failure was observed in the delayed surgery cases compared to the early surgery cases. Recently, other authors have reported that the time between trauma and reconstruction should be increased to 10 days to allow time for negative pressure wound therapy to improve the condition of the wound [22–24]. On the other hand, Alagöz *et al.* reported that early fluid fistula reconstruction should be performed in such cases [17] because the risk of meningitis was highest in the first week after CSF leakage [18]. Patients with contaminated wounds that involve the scalp, skull, and dura may be at greatest risk of developing meningitis [17,18]. Jimenez *et al.* reported on the risk of intracranial infection after a gunshot wound and analyzed the relationship between the length of time before treatment and the treatment outcome and concluded that early treatment was necessary to prevent posttraumatic meningitis [25]. We believe early reconstruction of dura and scalp defect using free tissue transfer could prevent meningitis.

In Case 1, the fascia lata was used for dura reconstruction, and the free rectus abdominis flap was used for soft tissue reconstruction. For Case 2, in contrast, a free anterolateral thigh flap with vascularized fascia lata was used for both the soft tissue and the dura reconstruction. Malliti *et al*. compared the use of synthetic dura with pedicled pericranium flaps for dura reconstruction and concluded that the rate of postoperative infection increased for dura reconstruction using synthetic material when compared to the pericranium flap [26]. There are no guidelines on whether the fascia should be vascularized or not, and we believe further study is required in this regard.

The reason why we could choose to perform emergency free tissue transfer in these cases was patients were healthy, they did not have any concomitant diseases preoperatively and meningitis may particularly decrease their quality of life. Our results suggest that emergency dura and soft tissue reconstruction using a free flap is viable and preferred in some cases, although a careful evaluation of the indications for this option should be performed.

## Conclusion

To prevent meningitis or leakage of cerebrospinal fluid in patients with scalp, skull, and dura defect after major trauma, the reconstructed dura should be covered with well-vascularized, bulky tissue. In such cases, free tissue transfer is useful and should be considered.
